# Transplacental transfer of SARS-CoV-2 antibodies: a cohort study

**DOI:** 10.1007/s10096-023-04553-5

**Published:** 2023-01-24

**Authors:** Stine Yde Nielsen, Lars Henning Petersen, May Murra, Lone Hvidman, Rikke Bek Helmig, Jens Kjølseth Møller, Mohammed Rohi Khalil, Maria Kirkeby, Tine Brink Henriksen

**Affiliations:** 1 Department of Clinical Microbiology, Lillebaelt Hospital, Vejle, University Hospital of Southern Denmark, Vejle, Denmark; 2 Department of Clinical Microbiology, Aarhus University Hospital, Aarhus, Denmark; 3 Biomedicine, Aarhus University, Aarhus, Denmark; 4 Department of Obstetrics and Gynecology, Aarhus University Hospital, Aarhus, Denmark; 5 Clinical Medicine, Aarhus University, Aarhus, Denmark; 6 Department of Obstetrics and Gynecology, Lillebaelt Hospital, University Hospital of Southern Denmark, Kolding, Denmark; 7 Child and Adolescent Medicine, Aarhus University Hospital and Clinical Institute, Aarhus University, Aarhus, Denmark

**Keywords:** SARS-CoV-2, Placental transfer ratio, Antibodies, Pregnancy, Infant, Neonatal

## Abstract

The purpose of this study was to examine the transfer rate of SARS-CoV-2 IgG antibodies in pregnancy and newborns. Two Danish labor wards screened all women for SARS-CoV-2 by PCR upon arrival. Women (*n* = 99) with a SARS-CoV-2 PCR–positive nasopharyngeal (NP) swab or with a household member with a positive swab at labor or any time during pregnancy, or COVID-19 symptoms upon admission (November 2020 through August 2021), were included. Mother and infant were tested by NP swabs at delivery, and maternal and infant (umbilical cord) venous blood samples were collected. We obtained clinical information including previous PCR test results from the medical records. SARS-Cov-2 IgM and quantified IgG antibodies were measured by enzyme-linked immunosorbent assay and transfer ratios of IgG. We detected IgG antibodies in 73 women and 65 cord blood sera and found a strong correlation between SARS-CoV-2 IgG concentrations in maternal and umbilical cord sera (*r* = 0.9; *p* < 0.05). Transfer ratio was > 1.0 in 51 out of 73 (69%) infants and > 1.5 in 26 (35%). We found that transfer was proportional to time from a positive SARS-CoV-2 PCR NP swab to delivery (*r* = 0.5; *p* < 0.05). Transfer ratios of SARS-CoV-2 antibodies were associated with time from infection to delivery with transfer ratios of more than 1.0 in the majority of seropositive mother-infant dyads.

## Introduction

The patterns of maternal antibody response to SARS-CoV-2 during pregnancy and degree of transplacental passage at various gestational ages are not well described. Serology at birth has shown a low seroprevalence in women during labor, but the majority of infants of seropositive mothers has detectable SARS-CoV-2 IgG antibodies at birth [1, 2].

Protection against early neonatal infection depends on the degree of transplacental passage of the infectious agent and transfer of maternal antibodies to the fetus. Transplacental transfer of maternal IgG may protect the neonate from infection. From the second trimester of pregnancy, IgG antibodies are usually passively transferred across the placenta from mother to fetus [3, 4]. Compared to maternal blood titers, umbilical cord titers of IgG have been reported to be approximately 1.5 times higher if IgG is induced by vaccination against diphtheria and pertussis [5, 6]. However, if induced by infection, transfer to the fetus of disease-specific antibodies during pregnancy may be lower, with a ratio of IgG closer to 1 [7, 8], but this ratio differs by the infectious agent.

In general, transfer of antibodies depends on several factors such as placental pathology, time elapsed from infection to delivery, gestational age at birth, maternal titers, and perhaps severity of illness during pregnancy, but also on nature of the antigen and properties of specific antibodies [3, 4, 9].

For SARS-CoV-2, evidence points to the rarity of transplacental transmission of the virus on one hand and on the other, there is increasing concern regarding compromised SARS-CoV-2-specific immunity in the neonate [10, 11].

The role of placental pathology in the setting of infection during pregnancy and whether placental involvement may alter the passage of antibodies per se need to be elucidated, and the relationship between timing of maternal infection, transfer ratio, and neonatal outcome is not well understood.

We therefore assessed quantitative SARS-CoV-2 IgG antibodies in mothers and infants and evaluated placental transfer and neonatal outcome by gestational age in maternal SARS-CoV-2 infection during pregnancy, and the time from infection to delivery.

## Methods

### Study population and sampling

We invited women admitted for delivery at Aarhus University Hospital and Lillebaelt Hospital from November 16, 2020, to August 23, 2021, to participate. Inclusion criteria were (1) COVID-19 symptoms upon admission, (2) a SARS-CoV-2 PCR–positive nasopharyngeal (NP) swab at delivery or any time during pregnancy, (3) a household contact who were SARS-CoV-2 PCR–positive by NP swab any time during the pregnancy, (4) COVID-19 symptoms in a household contact at the time of delivery, or (5) the ability to understand the oral and written information (in Danish).

From another, yet unpublished study by our group, all women who gave birth at Aarhus University and Lillebaelt Hospitals between April 23, 2020, and November 15, 2020, were invited to participate, irrespective of SARS-CoV-2 status or symptoms. From that study, we identified 17 seropositive mothers and their infants who were also included in the current study. Blood and swab sampling was identical in the two groups.

Upon admission, all women were screened for SARS-CoV-2 by a NP swab. Shortly before or immediately after delivery, a venous blood sample was drawn from the mother and a rectal and vaginal swab was performed. At the time of cord clamping, a blood sample was drawn from the umbilical vein, and a NP swab collected from the infant.

### PCR methods

PCR analysis was performed at Aarhus University Hospital with the Cobas® SARS-CoV-2 test by use of the Cobas® 6800 System (Roche Diagnostics, Indianapolis, USA) with detection of the ORF-1a/b and the E-gene or with an in-house PCR analysis with RNA extraction from samples and using the E-gene assay from the Charité protocol recommended by WHO [12]. Lillebaelt Hospital used the RealStar® SARS-CoV-2 RT-PCR Kit 1.0 (altona Diagnostics, Hamburg, Germany) for the detection of the E-gene (lineage B-beta coronavirus) and the S-gene (SARS-CoV-2-specific RNA).

Internal negative and positive controls were included for all PCR assays in both the purification step and in the real-time PCR step. All NP swab PCR analyses from the women were run within 24 h of sampling. PCR analyses from the plasma, rectum, vagina, and the infant NP swabs were performed after samples’ storage at + 5 °C for a maximum of 48 h. For women with more than one SARS-CoV-2 PCR–positive NP swab during pregnancy, only the first test was included in order to calculate time from infection to delivery.

### Antibody measurements

All serum samples were screened for SARS-CoV-2 total antibodies (Ab), i.e., IgG, IgM, and IgA, by use of Wantai’s commercial enzyme-linked immunosorbent assay (ELISA) (Wantai Biological Pharmacy Enterprise Co., Ltd., Beijing, China) which measures antibodies to the receptor binding domain of the spike protein, according to the manufacturer’s instructions.

The total Ab assay was based on a two-step incubation double-antigen sandwich principle, where the antigen is the SARS-CoV-2 receptor binding domain of the spike protein used for both coating and detection. *A* (absorbance)/CO (cut-off) values ≥ 1.0 were considered positive.

All samples were furthermore tested using the Wantai SARS-CoV-2 IgG (enzyme-linked immunosorbent) ELISA (Quantitative) solid-phase, indirect assay ELISA method for the detection of IgG-class antibodies to SARS-CoV-2 in a two-step incubation procedure. Microwell strips were pre-coated with SARS-CoV-2 recombinant antigens. During the first incubation step, SARS-CoV-2 IgG antibodies would bind to the solid-phase pre-coated antigens. Afterwards, anti-human IgG antibodies (anti-IgG) conjugated to horseradish peroxidase (HRP-conjugate) were added. During the second incubation step, these HRP-conjugated antibodies would bind to any antigen–antibody (IgG) complexes previously formed. In presence of the antigen–antibody-anti-IgG (HRP) immunocomplex, the absorbance value (*A* value) could be measured and was proportional to the titer of IgG antibody in the specimen.

Samples were diluted to be within the dynamic range of the assay (considered positive between 1.0 and 16.0 U/mL) and concentrations were calculated based on dilution factors.

All total Ab-positive samples were also tested for IgM using Wantai’s IgM assay. This is a two-step solid-phase antibody capture ELISA, using antibodies directed against the human IgM proteins and recombinant SARS-CoV-2 antigen. For the IgM assay, *A*/CO values ≥ 1.1 were considered positive and *A*/CO values < 0.9 were considered negative. *A*/CO values between these two values were considered inconclusive. We retested all samples with an *A*/CO value ≥ 0.9. No discrepancies were observed when retested and only positive samples were included in the analysis.

Information on maternal characteristics, pregnancy complications, infant characteristics, and conditions at delivery were retrieved from the electronic medical patient records by cross-referencing mother and infant by use of their unique personal identification number given to all Danish citizens at birth. Furthermore, all women were subjected to a structured interview by a midwife to collect information on all household members’ current and previous COVID-19 symptoms upon arrival to the labor ward.

### Statistical analysis

We used Fisher’s exact test for categorical variables due to few outcomes and the Wilcoxon rank-sum test for continuous variables with two groups. Median values are presented with inter quartile range (IQR).

Antibody concentrations were log-transformed and reported as geometric mean concentrations.

Transfer ratios were calculated as infant IgG concentration divided by maternal IgG concentration.

The Pearson correlation coefficient was used to evaluate correlations between (1) infant and maternal IgG concentrations and (2) transfer ratio and time between PCR on NP swab and delivery (Fig. 2A, B).

Data was analyzed by the use of Stata version 16 (StataCorp, TX, USA).

## Results

**Fig. 1:**
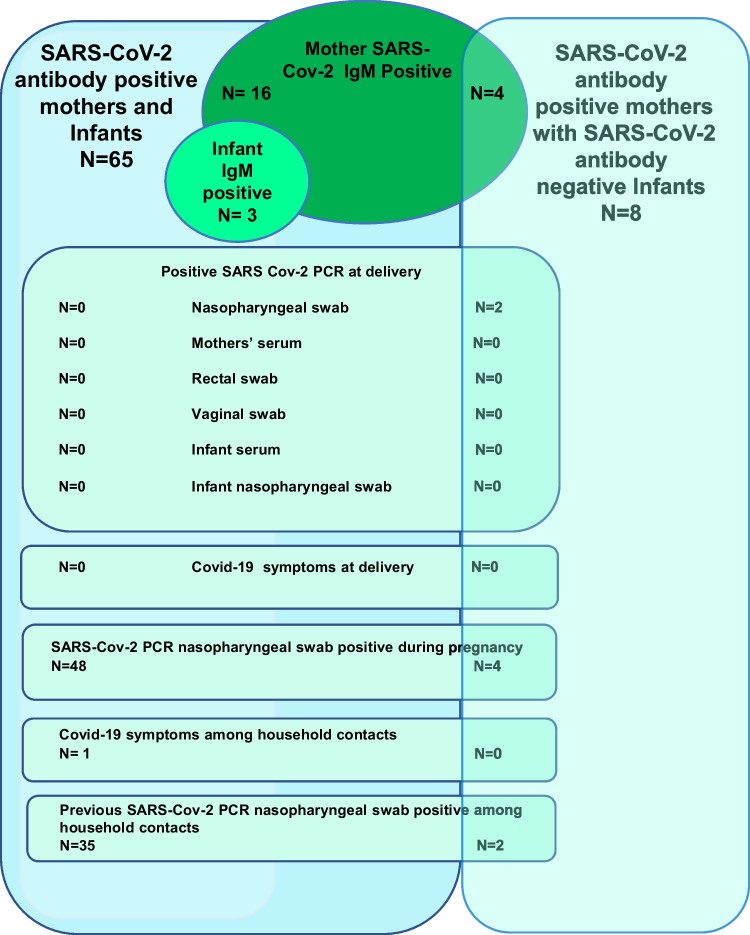
SARS-CoV-2 characteristics of 73 women and their infants, Aarhus University and Lillebaelt Hospitals

A total of 99 women fulfilled the inclusion criteria with available blood samples from both mother and infant. Of these, 26 women had not been infected but had a household contact who was SARS-CoV-2-positive during the pregnancy and were excluded, leaving 73 SARS-CoV-2 seropositive women eligible for analysis. Characteristics of women and their infants are presented in Table 1. PCR status and serology are presented in Fig. 1. None of the women was vaccinated during pregnancy and all were singleton pregnancies. No participating women had COVID-19 symptoms at delivery and we had no COVID-19-related admissions to the intensive care unit.

**Table 1:** Characteristics of SARS-CoV-2 seropositive (IgG) mothers (*n* = 73) and their infants at birth, Aarhus University and Lillebaelt Hospitals, Denmark

Mothers	Seropositive mother-infant dyads (*n* = 65)'	Seronegative mother-infant dyads (*n* = 8)''
Age, median (IQR)	31 (28–33)	30 (25–35)
BMI (kg/m^2^), median (IQR)	23.1 (20.7–26.0)	22.8 (22.1–30.0)
Smoking	1 (1.5%)	0
Maternal chronic disease^	1 (1.5%)	0
Pregnancy complications		
Gestational diabetes	3 (4.6%)	0
Gestational hypertension	5 (7.6%)	0
Preeclampsia	2 (3.1%)	0
Delivery		
Gestational age at delivery, median(IQR); weeks:days	40 (39:1–40:6)	40 (39:1–40:6)
Preterm birth (< 37 weeks)	1 (1.5%)	0
Cesarean delivery	12 (19.0%)	0
Antibiotics during delivery	15 (23.1%)	2 (25.0%)
SARS-CoV-2-related outcomes		
Mothers		
Serology (quantitative IgG)		
- IgG concentration geometric mean (95%CI)	3.4 (2.7–4.9)	3.0 (1.9–4.2)
Serology(IgM)		
- Positive	16 (24.2%)	4 (50.0%)
- Negative	49 (75.4%)	4 (50.0%)
COVID-19 symptoms at delivery	0	0
COVID-19 symptoms household contact	1 (1.5%)	0
Household contact SARS-Cov-2 PCR–positive > 7 days prior to delivery	35 (54.6%)	2 (25.0%)
Nasopharyngeal swab positive during pregnancy	48 (74.2%)	4 (50.0%)
Time between nasopharyngeal swab positive and delivery (median, IQR, days)	136 (79–170)	28 (21–41)
SARS-Cov-2 typing nasopharyngeal swab		
- Subtypes of SARS-CoV-2 *	8 (16.3%)	1 (16.8%)
- B.1.1.7 *	7 (14.3%)	1 (16.8%)
- B1.1617.2 *	0	0
- Other *	1 (2.0%)	0
SARS-CoV-2 PCR–positive at delivery	0	2 (25.0%)
- Nasopharyngeal swab**	-	2 (100%)
- Plasma	-	0
- Vaginal and rectal swab	-	0
Infants		
Serology (quantitative IgG)		
- IgG concentration geometric mean (95%CI)	4.0 (3.2–5.3)	-
Serology(IgM)		
- Positive	3 (4.6%)	0
- Negative	62 (95.4%)	8 (100%)
SARS-CoV-2-positive PCR immediately upon delivery		
- Cord serum and nasopharyngeal swab	0	0

'Seropositive mothers with seropositve infants

''Seropositive mothers with seronegative infants

^Thyroid disease

^*^Percentages among all positive NP PCR

^**^Percentages among PCR-positive at birth

All infants were born at 32 gestational weeks or later with birth weights of more than 2500 g, except for one infant (Table 2).

**Table 2:** Transfer ratio categories and infant characteristics of SARS-CoV-2 seropositive (quantitative IgG) mothers (*n* = 73) and their infants

	No transfer (*n* = 8)	Transfer ratio* < 0.5 (*n* = 2)	Transfer ratio* 0.5– < 1.0 (*n* = 12)	Transfer ratio* 1.0– < 1.5 (*n* = 25)	Transfer ratio* 1.5– < 2.0 (*n* = 14)	Transfer ratio* 2.0– < 2.5 (*n* = 5)	Transfer ratio* > = 2.5 (*n* = 7)
Preterm birth (< 37 weeks)	0	0	0	1 (4.0%)	0	0	0
Birth weight							
> = 2500 g	8 (100%)	2 (100%)	12 (100%)	24 (96.0%)	14 (100%)	5 (100%)	7 (100%)
≥ 1500– ≥ 2500 g	0	0	0	1 (4.0%)	0	0	0
Apgar score < 5 at 5 min	0	0	0	0	0	0	0
Maternal serology (quantitative IgG)							
IgG concentration geometric mean (95% CI)	3–0 (1.9–4.2)	6.0 (5.1–7.0)	5.1 (4.2–6.4)	3.43 (2.8–4.6)	3.1 (2.0–3.4)	2.7 (2.3–3.0)	3.9 (1.7–5.1)
Maternal IgM							
- Positive	4 (50.0%)	2 (100%)	5 (41.7%)	6 (24.0%)	1 (7.1%)	0	2 (28.6%)
- Negative	4 (50.0%)	0	7 (58.3%)	19 (76.0%)	13 (92.9%)	5 (100%)	5 (71.4%)
Mother NP positive during pregnancy	4 (50.0%)	1 (50.0%)	4 (33.3%)	5 (20.0%)	3 (21.4%)	1 (20.0%)	3 (42.9%)
- Time between—NP PCR pos and delivery	28 (21–41)	30 (30–30)	42 (35–136)	109 (75–153)	168 (142–246)	173 (127–216)	126 (114–150)
Infant IgM							
- Positive	0	0	3 (25.0%)	0	0	0	0
- Negative	8 (100%)	2 (100%)	9 (75.0%)	25 (100%)	14 (100%)	5 (100%)	7 (100%)

At delivery, only two women had a positive SARS-CoV-2 PCR NP swab and no woman reported symptoms.

A total of 65 (89%) of the seropositive women gave birth to a seropositive infant (seropositive dyads). Sixteen of these mothers were also IgM-positive. Eight women (11%) gave birth to a seronegative infant. Four of these mothers were IgM-positive.

Among the seropositive dyads, no woman had a SARS-CoV-2 PCR–positive NP swab at delivery. Among these, 49 women and 36 of their household contacts had a SARS-CoV-2 PCR–positive NP swab more than 7 days prior to delivery (Fig. 1).

All SARS-CoV-2 PCR analyses on maternal blood, vaginal, and rectal swabs were negative.

Among the seropositive dyads, three infants were IgM-positive. SARS-CoV-2 PCR analysis of the umbilical cord plasma and NP swabs were negative in all infants, irrespective of their antibody status, and there were no significant differences in obstetric complications or gestational age at birth between seropositive dyads and seropositive mothers with seronegative infants.

Transfer ratios by infant characteristics, maternal serology, and time between first maternal SARS-CoV-2 PCR–positive NP swab and delivery are presented in Table 2.

In 51 (69%) out of all the 73 seropositive women, the transfer ratio was > 1.0 and in 26 (35%) women, it was > 1.5.

**Fig. 2:**
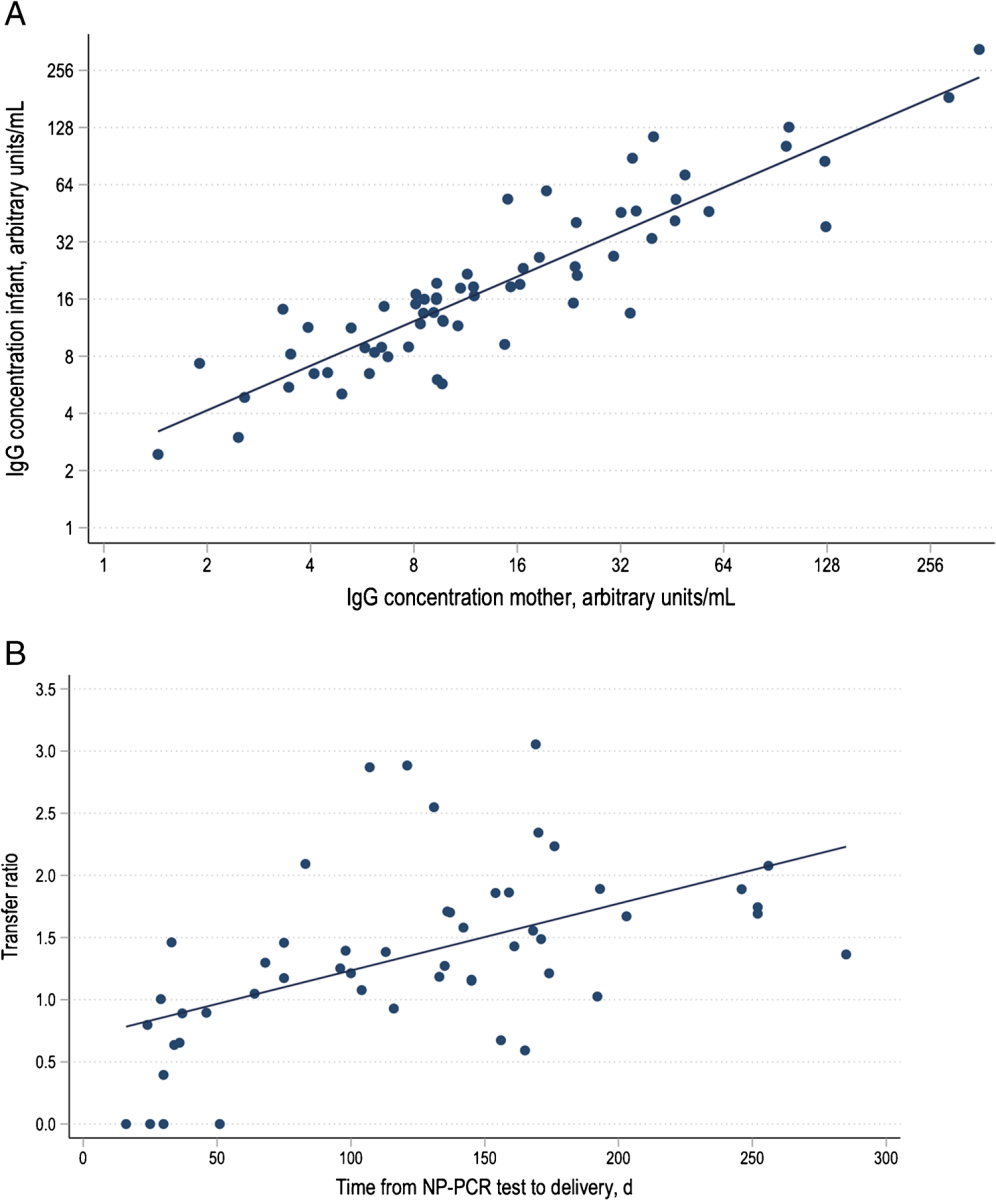
**A** Correlation between IgG concentrations in seropositive women and matched cord blood from 65 infants taken at delivery. **B** Association between days from SARS-CoV-2 NP PCR test to delivery and transplacental antibody transfer

For three IgM-positive infants, transfer ratios were between 0.5 and 1.0. A total of 11 of the 20 IgM-positive mothers had transfer ratios < 1.0 with four having no transfer. As shown in Fig. 2A, we found a strong correlation between SARS-CoV-2 IgG concentrations in maternal and umbilical cord sera (*r* = 0.9; *p* < 0.05) and that higher maternal antibody titers were associated with lower cord blood titers.

Among the seropositive mothers with seronegative infants, four mothers were IgM-positive; there was no association between maternal IgM status and transfer ratio of IgG.

For seropositive dyads, the median time between SARS-CoV-2 PCR–positive NP swab and delivery was 136 (83–170) days. For seropositive mothers with seronegative infants, the median time between the first positive SARS-CoV-2 PCR NP swab and delivery was 28 (21–41) days (Table 1).

As shown in Fig. 2B, the correlation between time from SARS-CoV-2 PCR–positive NP swab to delivery and transfer ratio (*r* = 0.5; *p*-value < 0.05) was moderate. Transfer ratio was > 1 in a majority mother-infant dyads with a first maternal SARS-CoV-2-positive NP swap 50 days or more prior to delivery.

## Discussion

### Main findings

We estimated SARS-CoV-2 total antibodies and quantitative IgG and IgM and carried out meticulous SARS-CoV-2 PCR testing of plasma and swabs from various anatomical locations in Danish women at the time of delivery. We detected IgG antibodies in 73 women and 65 cord blood sera. Our findings provide insight to the efficacy of placental antibody transfer of SARS-CoV-2.

We found a strong correlation between mother and infant IgG titers at birth. A transfer ratio below 1 in 14 (19%), of more than 1.0 in 51 (69%) of infants of 73 seropositive mothers, and more than 1.5 in 26 (35%) compared to no transfer in 8 (11%) indicate effective transfer of SARS-CoV-2 antibodies. Transfer ratios increased with the interval from maternal infection to delivery; the majority had transfer ratios more than 1.0 if delivery occurred 50 days or more after the infection. There was no indication of SARS-CoV-2 transmission from mother to infant based on meticulous SARS-CoV-2 PCR testing of infant plasma and NP swabs, even in IgM-positive neonates. Since SARS-CoV-2 PCR in umbilical cord blood and NP swabs from the IgM-positive infants were negative and keeping in mind the unreliability of IgM antibodies [13], these samples may have been false-positive, or perhaps contaminated by maternal blood.

### Strengths and limitations

The strengths of the present study include exhaustive SARS-CoV-2 PCR testing of seropositive women including blood, vaginal and rectal swabs, umbilical cord blood, and infant NP swabs immediately after delivery. Another strength is the use of the Wantai assays. This assay has been shown to be superior in total antibody and IgM assays in the comparison of 16 serological assays in a large, population-based Danish study (total antibody: sensitivity 96.7, specificity 99.5; IgM: sensitivity 82.7, specificity 99.0) and the quantitative IgG assay has a documented sensitivity of 98% [14, 15]. Another strength is the definite diagnosis of SARS-CoV-2 PCR infection by NP swabs. It is a strength that the participants were included from all three trimesters of pregnancy during the pandemic, that none of the participants was vaccinated, and that none was infected prior to becoming pregnant.

Our limitations were the small sample size which, however, may be counterbalanced by the meticulous sampling and testing. We could not control for factors such as placental integrity which may influence IgG transfer, and we were also unable to describe antibody transfer in infants born preterm less than 32 gestational weeks at birth.

There is a possibility that two patients from our study were also included in an earlier study of COVID-19 in pregnancy among Danish women [16].

### Interpretation

Our findings are in line with those of Flannery et al. who found that SARS-CoV-2 antibodies were transferred in 87% of seropositive mothers with a placental transfer ratio around 1.0 [1]. Kubiak et al. [17] also found that higher maternal titers were associated with lower cord blood titers, whereas Flannery et al. found that higher maternal antibody titers were associated with higher cord blood titers [1]. Proof of placental transfer of SARS-CoV-2 antibodies has been established, but uncertainties remain as to whether infant (umbilical cord) titers are higher than maternal titers since recent studies have reported transfer ratios at lower levels than ours, e.g., 0.8 [18] and 0.7 (8) [17].

The differences in association between maternal and cord titers and reported transfer ratios may depend on duration between infection and delivery as well as gestational age at delivery, which may explain these differences.

Generally, recent studies suggest that antibody Fc glycosylation may impact IgG transfer across the placenta [3, 4]. Two studies have demonstrated less efficient transfer of SARS-CoV-2 IgG compared to transfer of antibodies generated from infections by other vaccine-preventable agents such as influenza and pertussis. Edlow et al. [10] evaluated 77 mother-infant dyads and found that the transfer ratio of SARS-CoV-2 IgG was lower (0.72) than transfer of influenza antibodies (1.44) and concluded that SARS-CoV-2 antibody transfer was inefficient. By use of serology to characterize the Fc profile for specific antibodies against influenza, pertussis, and SARS-CoV-2 transfer across placenta, Atyeo et al. [11] also found that in the third trimester, antibody transfer ratios were lower for SARS-CoV-2 antibodies than for influenza virus and pertussis antibodies. Comparison of SARS-CoV-2 antibody response after infection with studies on vaccine data may be compromised by persistence and level of vaccine antibodies and by time between vaccination and delivery as well as gestational age at delivery.

Placental studies from women with SARS-CoV-2 during pregnancy have reported a characteristic triad of trophoblast necrosis, inflammatory intervillous infiltrates, and increased perivillous fibrinoid deposition along with a positive immunohistochemistry for SARS-CoV-2 antigens [19, 20]. These findings are from pregnancies with a SARS-CoV-2 immunohistochemistry–positive placenta presenting with signs of fetal distress or death after a SARS-CoV-2 infection during pregnancy but without transmission of virus to the fetus. These adverse pregnancy outcomes may primarily be caused by pathological effects of the virus with placental inflammation and insufficiency, but without the virus being found in the fetus or the newborn.

Accordingly, the less efficient transfer of SARS-CoV-2 IgG in at least some pregnancies may be due to infection-related changes in the placenta. However, our findings support the results from a few other studies showing that timing is an important factor as transfer ratios increase with time from infection to delivery, and that transfer may be insufficient if infection occurs close to the time of delivery [1, 21].

In a large, multicenter cohort study based on information from health records, Piekos et al. [22] found that COVID-19 severity was unrelated to the severity of pregnancy outcome. None of the women in our cohort suffered from severe COVID-19 infection, and there were no COVID-19-related admissions to intensive care unit. The median time from infection in pregnancy to delivery was 136 days for seropositive dyads and 28 for seronegative dyads and all but one infant were term born.

The SARS-CoV-2 BNT162b2 mRNA vaccine has been estimated to be safe throughout pregnancy [23] and to have high vaccine effectiveness in pregnant women [24]. A neonatal humoral response has been established [25–27] with transfer ratios varying according to timing of vaccination during the course of pregnancy [25, 26, 28]. Whether infection, vaccination, or treatment with monoclonal IgG antibodies in early pregnancy may prevent compromised placental perfusion and provide sufficient humoral immunity to SARS-CoV-2 in the infant is still unknown.

More studies are warranted to fully understand the ideal timing for vaccination of pregnant women, also with respect to gestational age at delivery, but also to clarify the level of IgG at which the immunity in the infant will be sufficient to prevent potential early-onset infection in the newborn.

## Conclusion

We found a strong correlation between mother and infant IgG titers at birth. Transfer ratio of SARS-CoV-2 antibodies was higher with the time elapsed from the infection to delivery with transfer ratios of more than 1.0 in the majority of seropositive mother-infant dyads. This may indicate that timing of infection, and potentially also of vaccination during pregnancy, may be important in order to secure immunity of the infant.

## Data Availability

The datasets generated during the current study are available from the corresponding author on reasonable request.

## References

[1] Flannery DD, Gouma S, Dhudasia MB, Mukhopadhyay S, Pfeifer MR, Woodford EC (2021). Assessment of maternal and neonatal cord blood SARS-CoV-2 antibodies and placental transfer ratios. JAMA Pediatr.

[2] Egerup P, Fich Olsen L, Christiansen AH, Westergaard D, Severinsen ER, Hviid KVR (2021). Severe acute respiratory syndrome coronavirus 2 (SARS-CoV-2) antibodies at delivery in women, partners, and newborns. Obstet Gynecol.

[3] Fouda GG, Martinez DR, Swamy GK, Permar SR (2018). The impact of IgG transplacental transfer on early life immunity. Immunohorizons.

[4] Jennewein MF, Abu-Raya B, Jiang Y, Alter G, Marchant A (2017). Transfer of maternal immunity and programming of the newborn immune system. Semin Immunopathol.

[5] Munoz FM, Bond NH, Maccato M, Pinell P, Hammill HA, Swamy GK (2014). Safety and immunogenicity of tetanus diphtheria and acellular pertussis (Tdap) immunization during pregnancy in mothers and infants: a randomized clinical trial. JAMA.

[6] Heininger U, Riffelmann M, Leineweber B, von Wirsing KCH (2009). Maternally derived antibodies against Bordetella pertussis antigens pertussis toxin and filamentous hemagglutinin in preterm and full term newborns. Pediatr Infect Dis J.

[7] Perret C, Chanthavanich P, Pengsaa K, Limkittikul K, Hutajaroen P, Bunn JE (2005). Dengue infection during pregnancy and transplacental antibody transfer in Thai mothers. J Infect.

[8] Castanha PMS, Souza WV, Braga C, Araujo TVB, Ximenes RAA, Albuquerque M (2019). Perinatal analyses of Zika- and dengue virus-specific neutralizing antibodies: a microcephaly case-control study in an area of high dengue endemicity in Brazil. PLoS Negl Trop Dis.

[9] Palmeira P, Quinello C, Silveira-Lessa AL, Zago CA, Carneiro-Sampaio M (2012). IgG placental transfer in healthy and pathological pregnancies. Clin Dev Immunol.

[10] Edlow AG, Li JZ, Collier AY, Atyeo C, James KE, Boatin AA (2020). Assessment of maternal and neonatal SARS-CoV-2 viral load, transplacental antibody transfer, and placental pathology in pregnancies during the COVID-19 pandemic. JAMA Netw Open.

[11] Atyeo C, Pullen KM, Bordt EA, Fischinger S, Burke J, Michell A (2021). Compromised SARS-CoV-2-specific placental antibody transfer. Cell.

[12] Corman VM, Landt O, Kaiser M, Molenkamp R, Meijer A, Chu DK et al (2020) Detection of 2019 novel coronavirus (2019-nCoV) by real-time RT-PCR. Euro Surveill 25(3):2000045. 10.2807/1560-7917.ES.2020.25.3.2000045 10.2807/1560-7917.ES.2020.25.3.2000045 PMC6988269 31992387

[13] Latiano A, Tavano F, Panza A, Palmieri O, Niro GA, Andriulli N (2021). False-positive results of SARS-CoV-2 IgM/IgG antibody tests in sera stored before the 2020 pandemic in Italy. Int J Infect Dis.

[14] Harritshøj LH, Gybel-Brask M, Afzal S, Kamstrup PR, Jørgensen CS, Thomsen MK et al (2021) Comparison of 16 serological SARS-CoV-2 immunoassays in 16 clinical laboratories. J Clin Microbiol 59(5):e02596–20. 10.1128/JCM.02596-20 10.1128/JCM.02596-20 PMC8091860 33574119

[15] Weidner L, Gansdorfer S, Unterweger S, Weseslindtner L, Drexler C, Farcet M (2020). Quantification of SARS-CoV-2 antibodies with eight commercially available immunoassays. J Clin Virol.

[16] Aabakke AJM, Krebs L, Petersen TG, Kjeldsen FS, Corn G, Wojdemann K et al (2021) SARS-CoV-2 infection in pregnancy in Denmark-characteristics and outcomes after confirmed infection in pregnancy: a nationwide, prospective, population-based cohort study. Acta Obstet Gynecol Scand 100(11):2097–2110. 10.1111/aogs.14252 10.1111/aogs.14252 PMC8652723 34467518

[17] Kubiak JM, Murphy EA, Yee J, Cagino KA, Friedlander RL, Glynn SM (2021). Severe acute respiratory syndrome coronavirus 2 serology levels in pregnant women and their neonates. Am J Obstet Gynecol.

[18] Joseph NT, Dude CM, Verkerke HP, Irby LS, Dunlop AL, Patel RM (2021). Maternal antibody response, neutralizing potency, and placental antibody transfer after severe acute respiratory syndrome coronavirus 2 (SARS-CoV-2) infection. Obstet Gynecol.

[19] Schwartz DA, Avvad-Portari E, Babál P, Baldewijns M, Blomberg M, Bouachba A et al (2022) Placental tissue destruction and insufficiency from COVID-19 causes stillbirth and neonatal death from hypoxic-ischemic injury: a study of 68 cases with SARS-CoV-2 placentitis from 12 countries. Arch Pathol Lab Med 146(6):660–676. 10.5858/arpa.2022-0029-SA 10.5858/arpa.2022-0029-SA 35142798

[20] Dubucs C, Groussolles M, Ousselin J, Sartor A, Van Acker N, Vayssière C (2022). Severe placental lesions due to maternal SARS-CoV-2 infection associated to intrauterine fetal death. Hum Pathol.

[21] Song D, Prahl M, Gaw SL, Narasimhan SR, Rai DS, Huang A (2021). Passive and active immunity in infants born to mothers with SARS-CoV-2 infection during pregnancy: prospective cohort study. BMJ Open.

[22] Piekos SN, Roper RT, Hwang YM, Sorensen T, Price ND, Hood L (2022). The effect of maternal SARS-CoV-2 infection timing on birth outcomes: a retrospective multicentre cohort study. Lancet Digit Health.

[23] Favre G, Maisonneuve E, Pomar L, Winterfeld U, Daire C, Martinez de Tejada B (2022). COVID-19 mRNA vaccine in pregnancy: results of the Swiss COVI-PREG registry, an observational prospective cohort study. Lancet Reg Health Eur.

[24] Dagan N, Barda N, Biron-Shental T, Makov-Assif M, Key C, Kohane IS (2021). Effectiveness of the BNT162b2 mRNA COVID-19 vaccine in pregnancy. Nat Med.

[25] Kugelman N, Nahshon C, Shaked-Mishan P, Cohen N, Sher ML, Gruber M et al (2022) Maternal and neonatal SARS-CoV-2 immunoglobulin G antibody levels at delivery after receipt of the BNT162b2 messenger RNA COVID-19 vaccine during the second trimester of pregnancy. JAMA Pediatr 176(3):290–295. 10.1001/jamapediatrics.2021.5683 10.1001/jamapediatrics.2021.5683 PMC8693209 34932066

[26] Beharier O, Plitman Mayo R, Raz T, Nahum Sacks K, Schreiber L, Suissa-Cohen Y et al (2021) Efficient maternal to neonatal transfer of antibodies against SARS-CoV-2 and BNT162b2 mRNA COVID-19 vaccine. J Clin Invest 131(19):e154834. 10.1172/JCI154834 10.1172/JCI154834 PMC8483743 34596052

[27] Nir O, Schwartz A, Toussia-Cohen S, Leibovitch L, Strauss T, Asraf K (2022). Maternal-neonatal transfer of SARS-CoV-2 immunoglobulin G antibodies among parturient women treated with BNT162b2 messenger RNA vaccine during pregnancy. Am J Obstet Gynecol MFM.

[28] Rottenstreich A, Zarbiv G, Oiknine-Djian E, Vorontsov O, Zigron R, Kleinstern G et al (2022) Timing of SARS-CoV-2 vaccination during the third trimester of pregnancy and transplacental antibody transfer: a prospective cohort study. Clin Microbiol Infect 28(3):419–425. 10.1016/j.cmi.2021.10.003 10.1016/j.cmi.2021.10.003 PMC8563509 34740773

